# Variant rs1801157 in the 3’UTR of SDF-1ß Does Not Explain Variability of Healthy-Donor G-CSF Responsiveness

**DOI:** 10.1371/journal.pone.0121859

**Published:** 2015-03-24

**Authors:** Miriam Schulz, Darja Karpova, Gabriele Spohn, Annette Damert, Erhard Seifried, Vera Binder, Halvard Bönig

**Affiliations:** 1 German Red Cross Blood Service Baden-Württemberg-Hesse, Frankfurt, Germany; 2 Institute for Transfusion Medicine and Immunohematology, Goethe University, Frankfurt, Germany; 3 Department of Pediatric Oncology, Hematology and Clinical Immunology, Medical Faculty, University of Duesseldorf, Duesseldorf, Germany; 4 University of Washington, Department of Medicine, Division of Hematology, Seattle, WA, United States of America; University-Hospital of Parma, ITALY

## Abstract

The genetics responsible for the inter-individually variable G-CSF responsiveness remain elusive. A single nucleotide polymorphism (SNP) in the 3’UTR of CXCL12, rs1801157, was implicated in X4-tropic HiV susceptibility and later, in two small studies, in G-CSR responsiveness in patients and donors. The position of the SNP in the 3’UTR together with *in-silico* predictions suggested differential binding of micro-RNA941 as an underlying mechanism. In a cohort of 515 healthy stem cell donors we attempted to reproduce the correlation of the CXCL12 3’UTR SNP and mobilization responses and tested the role of miR941 in this context. The SNP was distributed with the expected frequency. Mobilization efficiency for CD34+ cells in WT, heterozygous and homozygous SNP individuals was indistinguishable, even after controlling for gender. miR941 expression in non-hematopoietic bone marrow cells was undetectable and miR941 did not interact with the 3’ UTR of CXCL12. Proposed effects of the SNP rs1801157 on G-CSF responsiveness cannot be confirmed in a larger cohort.

## Introduction

Allogeneic G-CSF mobilized peripheral blood stem/progenitor cells from healthy volunteer donors have become the source of choice for allogeneic “stem cell” transplantation.[1–3] A hundred-fold difference in G-CSF responsiveness between poorly and well mobilizing donors was noticed early on, but except for an arguable, if anything weak effect of gender no predictors of mobilization response have been identified.[4–6] Several examples suggest that mobilization response is genetically determined: Humans subjected to successive cycles of G-CSF responded with similar mobilization both times.[5] In mice, strain-specific differences in G-CSF responsiveness have been appreciated for decades.[7, 8] The responsible genes remain elusive, however, despite significant efforts. Recently three small studies on autologous donors or healthy volunteer donors suggested a correlation between a single-nucleotide polymorphism (SNP) in the 3’URT of CXCL12ß, rs1801157, and G-CSF mobilization efficiency, which promised to be a break-through discovery in this field.[9–11]

**Fig 1:**
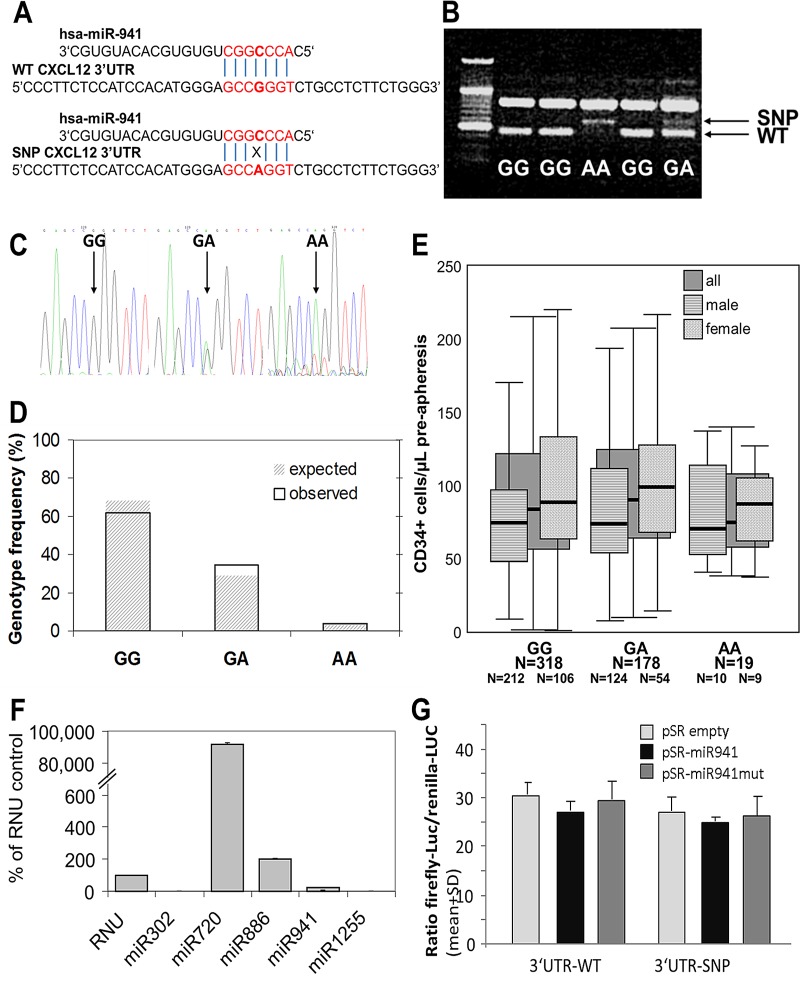
Variant rs1801157 of the SDF-1ß 3’UTR, miRNA941 and G-CSF responsiveness of healthy stem cell donors. **(A)** Sequence alignment of miR941 with WT- and SNP-variants of the 3’UTR of CXCL12. (**B)** Representative results from the sequence-specific PCR established to distinguish between WT- and SNP-variant. (**C)** Representative sequencing results from the 3’UTR genotyping. (**D)** Expected and observed frequencies of WT, heterozygous and homozygous SNP genotypes. (**E)** Circulating CD34+ cells after 9 doses q12h of G-CSF by CXCL12 3’UTR genotype. Mobilization efficiency for WT, heterozygous SNP and homozygous SNP donors was 94.9±2.9, 101.6±4.3 and 88.0±10.0 CD34+ cells/μl (mean±SEM), respectively. All donors combined are shown in dark grey bars, separate analyses by gender are overlaid. (**F)** miRNA expression in non-hematopoietic BM cells from healthy volunteer donors was tested by global miR expression arrays; individual highly and lowly expressed miRNAs were further tested by real-time PCR. miR941 expression was barely detectable. (**G)** The interaction between miR941 and the 3’UTR was assessed by dual luciferase assays. Luciferase activity was the same for the WT- and SNP-variant of the CXCL12 3’UTR, in the presence or absence of WT or mutated miR941. Hsa-miR1255 served as positive control for miRNA-mediated down-regulation of luciferase activity (not shown).

This SNP had previously been described to mediate susceptibility for X4-tropic strains of HiV.[12] Because of the great significance of this observation, it was carefully followed up and in more adequately-sized cohorts shown to be unreproducible.[13] Sparse and contradictory observations have been reported about effects of this SNP on CXCL12 production.[14–17] However, those reports and the position of SNP rs1801157 in the CXCL12 3’UTR suggested a possible influence of miRNAs, as these are known to mediate gene expression by binding to an mRNA’s 3’UTR. A seven bases long homologous sequence to the predicted seed of miR941 in the region of SNP rs1801157 was predicted by Target Scan Human (http://www.targetscan.org/) as a putative binding site, although this interaction was not predicted by any other microRNA web-tools. The 4^th^ base of this sequence being the polymorphic one, loss of the miRNA941 binding site of the SNP variant was predicted (Fig. 1A).

We therefore tested the correlation of SNP rs1801157 and G-CSF responsiveness in a representatively sized cohort of healthy volunteer donors. We also tested two prerequisites for miR941 mediated modulation of G-CSF induced mobilization, i.e. expression of miR941 in primary bone marrow (BM) stroma cells and differential interaction by miR941 with the WT- and SNP-variant of the CXCL12 3’UTR.

## Material and Methods

Healthy volunteer stem cell donors were subjected to G-CSF induced stem cell mobilization as described (7.5–10 μg/kgBW*d in two divided doses q12h),[18–20] in preparation for stem cell donation. Circulating CD34+ cells were enumerated 2–3 h after the ninth dose of G-CSF, just prior to apheresis, using commercial single-platform flow cytometry assays.[21] With written informed donor consent and permission from the Ethics Committee of Johann Wolfgang Goethe University School of Medicine (permit #190/12), DNA was isolated from pseudonymized left-over blood samples (Qiagen, Hilden, Germany). Circulating CD34+ cell concentration and gender were extracted from our stem cell donor data base. An SSP-PCR was established that distinguishes between WT- and SNP-variants (Fig. 1B). Samples were additionally genotyped for the SNP using Sanger sequencing (Fig. 1C), i.e. all genotyping analyses were performed with two independent methods. Mobilization data were sorted by 3’UTR genotype and gender. miR941 expression in non-hematopoietic BM stroma cells was tested in 20 anonymized CD45-purged BM samples from healthy volunteer donors using miR expression arrays (Miltenyi Biotec, Bergisch Gladbach, Germany) and taqMan qPCR (stem-loop RT primers and assay: Applied Biosystems, Darmstadt, Germany). miR941 binding to the WT- or SNP variant CXCL12 3’UTR was tested by cloning premiR941 (Eurofins, Hamburg, Germany) or SNP-premiR941 (premiR941 mutated in the 4^th^ position of the seed by site directed mutagenesis (Agilent technologies, Böblingen, Germany), to match the sequence of the SNP) into plasmid pSUPER.retro.puro (Oligoengine, Seattle, WA) and cloning of the whole 3’UTR of WT or SNP CXCL12ß (3285 bp) into the dual-luciferase plasmid pmirGLO (Promega, Mannheim, Germany). Plasmids were co-expressed in HEK 293 cells via lipofectamine (Life Technologies, Darmstadt, Germany). Transfection efficiency and expression of miR941 were tested by TaqMan qPCR (Applied Biosystems). Firefly and renilla luciferase activity were measured with the Dual-Glo Luciferase Assay System (Promega). In addition to miR941 expression by p.SUPER.retro.puro, synthetic miR941 and SNP-miR941 (Qiagen) were alternatively co-transfected with the pmirGLO 3’UTR variants. Cross-over analyses for WT- or SNP-3’UTR variant and WT- or mutated miR941were performed. Experiments were performed with commercial kits according to manufacturers’ instructions. Custom oligonucleotides are listed in Table 1. Descriptive statistics and t-tests (Student’s t-test or one-way ANOVA) were calculated using SPSS for Windows, version 11.0 (Statcon, Witzenhausen, Germany). A p<0.05 was considered statistically significant.

**Table 1:** Custom oligonucleotides used for these studies. PCR conditions are available from the authors upon request.

Primer name	Nucleotide sequence
SSP/Sequencing fwd	5‘ GTCAGCCCTAGGGTGGAGAG 3‘
SSP/Sequencing rev	5‘ CCTGCTTGGTGCACAGTTTA 3‘
SSP fwd	5‘ CATCCACATGGGAGCCA 3‘
SSP rev	5‘ TCCCAGAAGAGGCAGACCC 3‘
SNP miR941 fwd	5‘ GAAGAGGACGCACCTGGCTGTGTGCACAT 3‘
SNP miR941 rev	5‘ ATGTGCA CACAGCCAGGTGCGTCCTCTTC 3‘
3’UTR fwd	5‘ GAGCTCGAGGGTCAGACGCCTGAGGAAC 3‘
3’UTR rev	5‘ TCTAGATGCCCTGTTTTCCATGAACCACTGT 3‘

## Results and Discussion

These studies had been triggered by visibly published reports of SNP rs1801157 effects on CXCL12 protein levels and, likely in consequence thereof, on infectivity of X4-tropic HiV strains.[12, 14–16] These data were later refuted by appropriately-sized studies.[13] Nevertheless, two studies containing 63 patients [9] or 65 donors [10] suggesting effects of the SNP on HSPC mobilization were published. Samples from 515 consecutive donors, i.e. an eight-fold larger cohort than in above-referenced studies, were tested. Mobilization efficiency on the 5^th^ day of G-CSF was 97.0±2.4 CD34+ cells/μl (mean±SEM), in agreement with published data for split-dose G-CSF.[18–20] Distribution of genotypes was in agreement with expected frequencies (Fig. 1D). Mobilization was equally efficient in all genotypes (Fig. 1E). The same analyses were repeated after controlling for gender (Fig. 1E) and by analyzing together donors heterozygous and homozygous for the SNP (not shown), with the same outcome. Importantly, the first study connecting SNP rs1801157 with mobilization deals with patients undergoing autologous donation. By the time patients undergo mobilization and autologous stem cell collection, they have received considerable cumulative doses of myelotoxic chemotherapy.[22–24] Chemotherapy-induced mobilopathy due to impaired stem cell reserve is a well-recognized confounder of autologous mobilization responses far in excess of that of the genetically determined variability.[25] Moreover, given the role of the CXCL12/CXCR4 axis in stem cell maintenance, if indeed the SNP genotype was associated with altered CXCL12 expression in BM, then the same chemotherapy could cause different degrees of stem cell depletion depending on SNP genotype. Thus alternative hypotheses to explain the observations must be considered, although they are not raised, let alone explored by the authors.[9] Further autologous cohorts were not tested. Bogunia-Kubik et al. [10] claimed to support the notion of the SNP conveying a good-mobilizer phenotype in healthy volunteer stem cell donors. However, mobilization efficiency (CD34+ cells/μl) was not actually analyzed in that study. Instead, conclusions are based on the number of CD34+ cells in the apheresis product. In the study, men were two-fold overrepresented among the SNP carriers. Of relevance to the authors’ conclusions, gender has a very strong effect on apheresis outcomes: The two factors determining (and often limiting) blood throughput in donors are ACD-A infusion rate (≤1.2 ml/L total blood volume*min) and venous capacity. As a consequence, much greater blood flow rates and hence, apheresis process volumes are achieved in the (on average heavier) male. In donors with the same mobilization response (CD34+ cells/μL), this difference results, on average, in one-third greater stem cell harvests in men than in women (own unpublished data). Clearly, the observed differences in the parameters presented by Bogunia-Kubik et al.,[10] such as number of aphereses required to achieve target dose and CD34+ cell dose collected per kgBW, can be explained by this effect alone instead of effects of SNP rs1801157. Moreover, several studies report greater G-CSF responsiveness of males compared to females,[4, 6, 26] but this one as well as a previous one of ours does not.[5] In agreement with our data, analysis of a large donor cohort recently also showed no effect of the CXCL12 3’UTR genotype on mobilization.[6]

A putative binding site for hsa-miR-941 was predicted by Target Scan Human (http://www.targetscan.org/) in the region of SNP rs1801157 and loss thereof in the SNP variant was predicted. Even though none of several other commonly used miRNA search engines (Ensembl version 69, miRBase version 18, MICRORNA.ORG, MIRDB, RNA22-HSA, TARGETMINER, TARGETSCAN-VERT) predicted this interaction, to end the discussions about putative roles of SNP rs1801157 in mobilization responses, we explored whether the CXCL12 3’UTR polymorphism quantitatively affects mRNA expression and what is the role, if any, of miR941 in this context. For miR941 to differentially influence CXCL12 expression depending on the CXCL12 3’UTR genotype, the expectation would be that it must be expressed in BM stroma cells, the main source of CXCL12 in BM. miRNA expression arrays in non-hematopoietic BM cells, corroborated by qPCR, detected several highly expressed miRNAs, but no expression of miR941 (Fig. 1F). We also assessed (differential) association of miR941 with the SNP-region of the 3’UTR of CXCL12, as *in silico* analyses had suggested. Direct evidence was sought using dual luciferase assays. As we are showing, luciferase expression was not affected by co-expression of miR941, nor were differential effects on the different genotypes or differential effects of miR941 and mutant-miR941 observed (Fig. 1G). Linkage of SNP rs1801157 with other SNPs in the CXCL12 gene was not suggested by database searches. These data are in complete agreement with our observation that SNP rs1801157 does not affect mobilization responses.

In summary, our studies definitively contradict previous reports about effects of the SNP rs1801157 on G-CSF responsiveness in donors, just as previous reports of an effect of this polymorphism on susceptibility towards X4-tropic HI virus were unhinged when sufficiently large cohorts of HIV patients were analyzed.[13] We propose that unaccounted for confounders, poorly balanced within the very small cohorts, are responsible for the unreproducible data that were previously published.[9, 10] Of note, another study, similarly sized as ours, comes to the same conclusions.[6] The computer-predicted miRNA binding site in the CXCL12 3’UTR could not be confirmed, underscoring the necessity to experimentally confirm computer algorithm-predicted molecular interactions. The genetics underlying G-CSF responsiveness remain elusive.
